# Induction of Embryogenesis in *Brassica Napus* Microspores Produces a Callosic Subintinal Layer and Abnormal Cell Walls with Altered Levels of Callose and Cellulose

**DOI:** 10.3389/fpls.2015.01018

**Published:** 2015-11-25

**Authors:** Verónica Parra-Vega, Patricia Corral-Martínez, Alba Rivas-Sendra, Jose M. Seguí-Simarro

**Affiliations:** Cell Biology Group, COMAV Institute, Universitat Politècnica de ValénciaValencia, Spain

**Keywords:** androgenesis, benzyl alcohol, caffeine, doubled haploids, microspore embryogenesis, rapeseed, cell wall carbohydrates

## Abstract

The induction of microspore embryogenesis produces dramatic changes in different aspects of the cell physiology and structure. Changes at the cell wall level are among the most intriguing and poorly understood. In this work, we used high pressure freezing and freeze substitution, immunolocalization, confocal, and electron microscopy to analyze the structure and composition of the first cell walls formed during conventional *Brassica napus* microspore embryogenesis, and in cultures treated to alter the intracellular Ca^2+^ levels. Our results revealed that one of the first signs of embryogenic commitment is the formation of a callose-rich, cellulose-deficient layer beneath the intine (the subintinal layer), and of irregular, incomplete cell walls. In these events, Ca^2+^ may have a role. We propose that abnormal cell walls are due to a massive callose synthesis and deposition of excreted cytoplasmic material, and the parallel inhibition of cellulose synthesis. These features were absent in pollen-like structures and in microspore-derived embryos, few days after the end of the heat shock, where abnormal cell walls were no longer produced. Together, our results provide an explanation to a series of relevant aspects of microspore embryogenesis including the role of Ca^2+^ and the occurrence of abnormal cell walls. In addition, our discovery may be the explanation to why nuclear fusions take place during microspore embryogenesis.

## Introduction

Microspore embryogenesis is a fascinating experimental process whereby a haploid microspore is reprogrammed to become a haploid or doubled haploid (DH) embryo (Seguí-Simarro and Nuez, 2008a). This inducible pathway has a great biotechnological potential, since among many other advantages, it allows for faster and cheaper ways to obtain pure lines for hybrid seed production (Forster et al., 2007). In addition, it offers the possibility to be used as an *in vitro* model to study different basic and induced processes. Indeed, the androgenic switch is induced by the application of different types of abiotic stresses, including heat shock, cold, and starvation, among others (Shariatpanahi et al., 2006). Once induced, the cellular responses to abiotic stresses coexist with a developmental switch toward embryogenesis, and with the cessation of the old gametophytic program (Malik et al., 2007; Seguí-Simarro and Nuez, 2008a). Conceivably, all these changes must imply a profound remodeling at the genetic and molecular levels, and also in cell architecture. Among all the changes undergone by the embryogenic microspore, one of the aspects that attracted the attention of the first cell biologists that studied this process was how induced cells are divided (Zaki and Dickinson, 1991; Simmonds and Keller, 1999).

In somatic-type plant cells, the first structural marker of cell division is the microtubular pre-prophase band (PPB), which defines the future division plane (Pickett-Heaps and Northcote, 1966). By late anaphase phragmoplast initials are formed, and at early telophase, a tubulo-vesicular network (TVN) cell plate is assembled in the middle of a solid phragmoplast (Seguí-Simarro et al., 2004; Austin et al., 2005). At mid telophase, a ring-shaped transitional phragmoplast marks the transformation of the central region of the cell plate into a wide tubular network and then into a maturing, planar fenestrated sheet, while the actively growing peripheral zone expands centrifugally and eventually fuses with the mother cell wall. Finally, at late telophase the peripheral zone matures too, and the cell plate is transformed into a new cell wall (reviewed in Seguí-Simarro et al., 2008). These orchestrated changes in cell plate structure are accompanied by the deposition of different polysaccharides in a timely manner (reviewed in Worden et al., 2012; Drakakaki, 2015). The first polysaccharides present in the nascent cell plate would be pectins and hemicelluloses. Then, the synthesis of copious amounts of callose in the cell plate lumen is responsible for the transformation of the TVN cell plate into a maturing tubular network, and for the widening of these tubules into fenestrated sheets (Samuels et al., 1995). The final transformation of the planar fenestrated sheet-type cell plate into a new primary cell wall involves the progressive replacement of callose deposits by cellulose fibrils (Kakimoto and Shibaoka, 1992; Samuels et al., 1995; Otegui and Staehelin, 2000). Proper cellulose deposition appears essential for cell plate stabilization, as revealed by the aborted cell plates present in cellulose-deficient mutants (Zuo et al., 2000; Beeckman et al., 2002). Finally, cellulose combines with the already secreted hemicellulose molecules into a cellulose-hemicellulose network, while pectic polysaccharides reorganize to form the pectin-rich middle lamella (Carpita and McCann, 2000). In the final, somatic-type primary cell wall, callose is absent with the exception of the region around plasmodesmata, where it is supposed to play a regulatory role in cell-to-cell movement of molecules (Levy et al., 2007).

Based on this canonical pattern, some specialized cell types have developed alternative division mechanisms adapted to their function. This is the case, for example, of microspores. The first pollen mitosis (PMI) that transforms a microspore into a young pollen grain is characterized by the absence of a previous PPB (Van Lammeren et al., 1985), and by the building of an asymmetric phragmoplast (Brown and Lemmon, 1991), giving rise to the large, vegetative cell and the small, generative cell of the pollen grain. The cell wall formed around the generative cell is also special, since it is hemispherical and transiently rich in callose (Park and Twell, 2001). However, soon it was found that microspores committed to *in vitro* embryogenesis significantly diverge from these cell division patterns. First, embryogenic microspores usually divide symmetrically, as opposed to *in vivo* microspores, in a mechanism more similar to somatic-type cytokinesis than to PMI cytokinesis (Zaki and Dickinson, 1991). Accordingly, it was found that embryogenic microspores show the typical PPB of somatic-type dividing cells (Simmonds and Keller, 1999). The cell walls of induced microspores and microspore-derived embryos (MDEs) present other unique features. For example, it was recently shown that embryogenic microspores exhibit abundant deposits of excreted cytoplasmic material (Corral-Martínez et al., 2013). These walls are also characterized by altered levels of certain cell wall components, such as xyloglucans, pectins (Barany et al., 2010), and arabinogalactan proteins (El-Tantawy et al., 2013). The major cell wall polymer, cellulose, has also been investigated, but unfortunately, the results are somehow confusing. For example, Schulze and Pauls (2002) assumed that the first cell walls produced in *Brassica napus* embryogenic microspores are somatic-type and therefore, cellulose-rich. In olive, Solis et al. (2008) described the presence of cellulose in some embryogenic microspores, at both the inner cell walls and the *“thick peripheral wall localized below the exine.”* However, Dubas et al. (2013) recently demonstrated by calcofluor white staining that cellulose was absent from the first cell walls formed in few-celled *B. napus* embryogenic microspores, whereas older MDEs presented abundant cellulose signal in their walls.

The other major cell plate-forming polymer, callose, is a β-1,3-glucan essential not only for somatic-type cell plate formation, but also for the cellular response to biotic and abiotic stresses (reviewed in Stone and Clarke, 1992; Chen and Kim, 2009). During microspore development, the presence of callose as the main component of the tetrad cell walls appears critical for a proper development of the microspore, since rice *GSL5* knock-out mutants, defective in callose deposition during post-meiotic cytokinesis, exhibit a severe male-sterile phenotype (Shi et al., 2014). Later on, the deposition of a thin callose layer below the exine was proposed to have a role in exine patterning (Dong et al., 2005). In immature pollen grains, callose is found in the cell walls of the generative cell (Gorska-Brylass, 1967; Heslop-Harrison, 1968). Upon germination, callose progressively accumulates beneath the intine and is a major component of the pollen tube (Meikle et al., 1991; Ferguson et al., 1998). In the particular case of the first embryogenic divisions of the microspore, Telmer et al. (1995) found callose at the connections of the new cell wall with the mother cell wall, attributing it to alterations in the plasma membrane or the cell wall physiology. Other than that, little is known about the role of callose in the development of the MDE.

In this work we present a detailed study of the cell walls present in *B. napus* microspores during the initial stages of their induction toward embryogenesis. We analyzed a number of confocal three-dimensional series obtained from cultured microspores subjected to different experimental treatments, and a collection of transmission electron microscopy (TEM) images obtained from samples processed by high pressure freezing and freeze substitution (HPF-FS). HPF-FS is known as the best procedure to preserve even the most labile and/or transient ultrastructural elements of *in vivo* physiological processes (Gilkey and Staehelin, 1986), as well as during the androgenic switch (Corral-Martínez et al., 2013). Our samples covered all the stages of *B. napus* microspore embryogenesis, the different cell types formed, and the subcellular changes undergone as a consequence of embryogenesis induction. Together, our results shed light on the dynamics of callose and cellulose deposition during the initials of the androgenic switch, and its significance for further MDE development.

## Materials and Methods

### Plant Materials

*Brassica napus* L. donor plants of the highly embryogenic cv. Topas were grown as previously described (Seguí-Simarro et al., 2003). Plants were grown in the greenhouses of the COMAV Institute (Universitat Politècnica de València, Spain), the University of Colorado (Boulder, CO, USA), and the Plant Research International (Wageningen, The Netherlands).

### *Brassica napus* Microspore Cultures

Flower buds containing mostly vacuolated microspores were selected and processed as previously described (Custers, 2003). Briefly, buds were surface sterilized with 2% sodium hypochlorite for 10 min, and washed three times in sterile distilled water. To release the microspores, buds were gently crushed in filter sterilized NLN-13 medium with the back of the plunger of a disposable 50 ml syringe. NLN-13 medium consists of NLN medium (Lichter, 1982) + 13% sucrose. Then, the slurry was filtered through 30 μm nylon cloths. The filtrate was transferred to 15 ml conical tubes and centrifuged at 800 rpm for 4 min. After discarding the supernatant, the pellet of microspores was resuspended in 10 ml of fresh NLN-13 medium. This procedure was additionally repeated twice for a total of three centrifugations and resuspensions. Before the last centrifugation step, microspore concentration was calculated using a hemacytometer. The required volume of NLN-13 medium was added to adjust suspension to a concentration of 4 × 10^4^ microspores per ml. For caffeine and benzyl alcohol (BA) experiments, NLN-13 medium supplemented with 1 mM caffeine and 100 μM BA were used, respectively. Adjusted microspore suspension was distributed in sterile culture dishes. Dishes were incubated in darkness for 24 h at 32°C to induce embryogenesis, and then continuously at 25°C for MDE progression up to the time of sample collection and processing.

### Callose and Cellulose Staining for Confocal Laser Scanning Microscopy

Samples of *B. napus* microspore cultures were collected at day 1, 2, 3, 4, and 6 after isolation, and fixed with 4% paraformaldehyde in phosphate buffered saline (PBS) pH 7.4. For callose staining, samples were placed in glass slides with 0.9% agarose, and stained with 10 μg/ml propidium iodide (PI) in PBS for 10 min. PI was used in order to have a reference of the subcellular staining pattern of aniline blue. PI is a general stain for nucleic acids, binding both DNA and RNA. Thus, when a previous RNAse treatment is omitted, the cytoplasm is also stained with PI (Suzuki et al., 1997). After three washes with PBS, samples were stained with 0.1% aniline blue (Evans et al., 1984) dissolved in PBS for 20 min, and then washed with the same buffer.

For cellulose staining, we used two different fluorescent stains, Calcofluor White ST and Pontamine Fast Scarlet (S4B). Calcofluor White was used diluted to 0.05% in water (Dubas et al., 2013). PI was also used better identification of the developmental stage of the microspore as described above. Both stains were added directly to the fixed microspores and slides were mounted with Vectashield and kept in darkness for at least 15 min before microscopic analysis. For S4B staining, samples were stained with 0.01% S4B in PBS for 30 min (Anderson et al., 2010), then washed thrice with PBS, and finally mounted with a mix of Vectashield and DAPI (1.25 μg/ml according to Custers et al., 1994) in a 1:4 proportion. Double staining with aniline blue and S4B was also performed as described above but combining both stains at 0.05 and 0.01%, respectively. Finally, preparations were observed with a Leica CTR 5500 and a Zeiss LSM 780 confocal laser scanning microscopes. Digital 3-D series of images were processed with Leica Application Suite Advanced Fluorescence (LAS AF) and FIJI software.

### Processing of *B. napus* Anthers and Microspore Cultures for TEM

Anthers carrying microspores and pollen grains at different stages of microsporogenesis and microgametogenesis were excised, transferred to aluminum sample holders, cryoprotected with 150 mM sucrose, frozen in a Baltec HPM 010 high-pressure freezer (Technotrade, Manchester, NH, USA) and a Leica EM HPM-100 high-pressure freezer (Leica Microsystems, Vienna), and then transferred to LN_2_. Cultured microspores and small MDEs were recovered from culture dishes by gently spinning culture media. Larger MDEs were manually picked up from cultures. These samples were transferred to aluminum sample holders, cryoprotected with their same glucose-rich culture medium and high-pressure frozen. All samples were freeze substituted in a Leica AFS2 system (Leica Microsystems, Vienna) with 2% OsO_4_ in anhydrous acetone at –80°C for 7 days, followed by slow warming to room temperature over a period of 2 days. After rinsing in several acetone washes, they were removed from the holders, incubated in propylene oxide for 30 min, rinsed again in acetone, and infiltrated with increasing concentrations of Epon resin (Ted Pella, Redding, CA, USA) in acetone according to the following schedule: 4 h in 5% resin, 4 h in 10% resin, 12 h in 25% resin, and 24 h in 50, 75, and 100% resin, respectively. Polymerization was performed at 60°C for 2 days. Using a Leica UC6 ultramicrotome, thin sections (1 μm) were obtained for light microscopy observation, and ∼80 nm sections were obtained for electron microscopy. Sections were mounted on formvar-coated, 200 mesh copper grids, stained with uranyl acetate and lead citrate, and observed in a Philips CM10 electron microscope.

### Immunogold Labeling

For the immunodetection of callose we used an anti-callose, mouse IgG monoclonal antibody (Biosupplies, Australia) crossreacting with linear β-1,3-oligosaccharide segments in β-1,3-glucans (Meikle et al., 1991). Immunogold labeling was performed in HPF-fixed, OsO_4_-treated, epoxy-embedded samples. This type of processing is not usually employed for immunogold labeling because it may preclude the immunolocalization of protein epitopes. Notwithstanding this, we used these samples for immunolocalization because the epitope studied (callose) is a carbohydrate, insensitive to the effects of OsO_4_ and the heat used to polymerize the resin. This way, we were able to combine specific immunolabeling with the excellent ultrastructural preservation provided by the use of these methods. 80–100 nm sections were deposited on Formvar and carbon-coated, 200-mesh nickel grids. Sections were hydrated with distilled water for 1 min, 1x PBS for 1 min, and blocked with 5%BSA in PBS for 5 min. Then, sections were incubated for 1 h at 25°C with anti-callose antibody, diluted 1:5,000 in 1% BSA. Next, sections were subjected to three 4-min washes with PBS and incubated for 45 min at 25°C with a goat anti-mouse secondary antibody conjugated to 10 nm colloidal gold (BBI Solutions, UK), diluted 1:25 in 1% BSA. Then, sections were subjected to three 4-min washes with PBS and one with distilled water. Finally, sections were counterstained with 0.5% uranyl acetate in 70% methanol and lead citrate, 10 min each.

## Results

**FIGURE 1:**
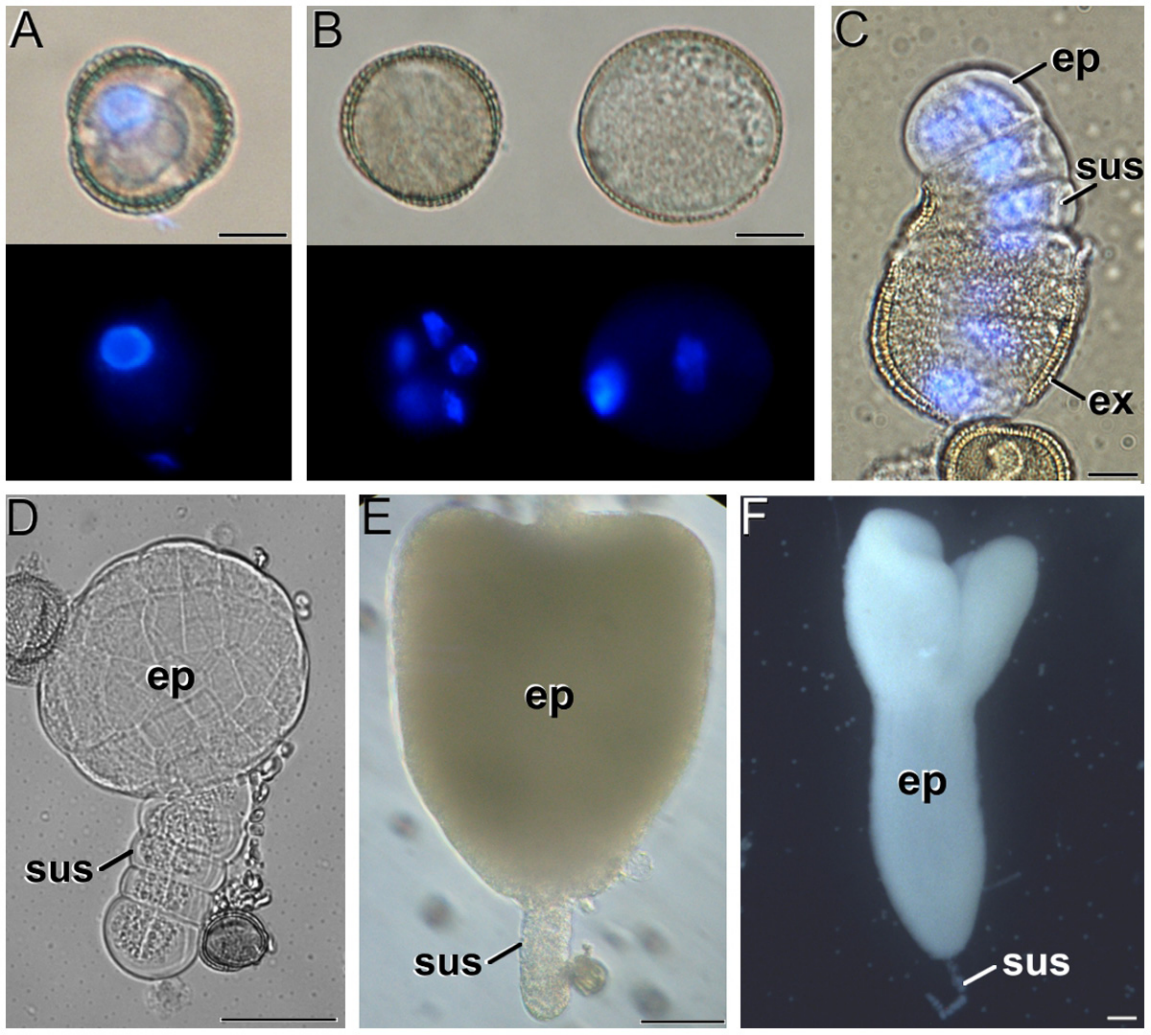
**The different stages of *Brassica napus* microspore cultures. (A)** Isolated microspore, prior to induction. **(B)** Induced, embryogenic microspore (left) and pollen-like structure (right). Top and bottom images in **(A,B)** were taken under phase contrast and fluorescence with DAPI-stained nuclei, respectively. Note the presence of up to five nuclei in the embryogenic microspore, and the presence of a brighter (generative) and a faintly fluorescing (vegetative) nucleus in the pollen-like structure. **(C)** Quadrant-like embryo emerging from the exine (ex), where the suspensor (sus) and the embryo proper (ep) domains can be clearly identified. Superimposed phase contrast + fluorescence image where the DAPI-stained nuclei are shown in blue. **(D)** Globular embryo. **(E)** Heart-shaped embryo. **(F)** Cotyledonar embryo. Bars in A–C: 10 μm; D: 50 μm; E, F: 100 μm.

In *B. napus* vacuolate microspores (**Figure 1A**), embryogenesis starts with a set of cell divisions still within the microspore exine (**Figure 1B**, left). In parallel, other microspores insensitive to induction may develop as pollen-like structures (**Figure 1B**, right) or just arrest and die. Successive divisions in embryogenic microspores give rise to exine rupture and to the emergence of young MDEs where the embryo proper and suspensor domains can soon be distinguished (**Figure 1C**). MDEs then follow a typical embryogenic pattern proceeding through the globular (**Figure 1D**), heart-shaped (**Figure 1E**), torpedo, and cotyledonar stages (**Figure 1F**). Samples of all the stages shown in **Figure 1** were collected at different culture stages and studied as follows.

### Embryogenic Microspores Occasionally Present Discontinuous, Incomplete Cell Plates, and Nuclear Fusion Profiles

**FIGURE 2:**
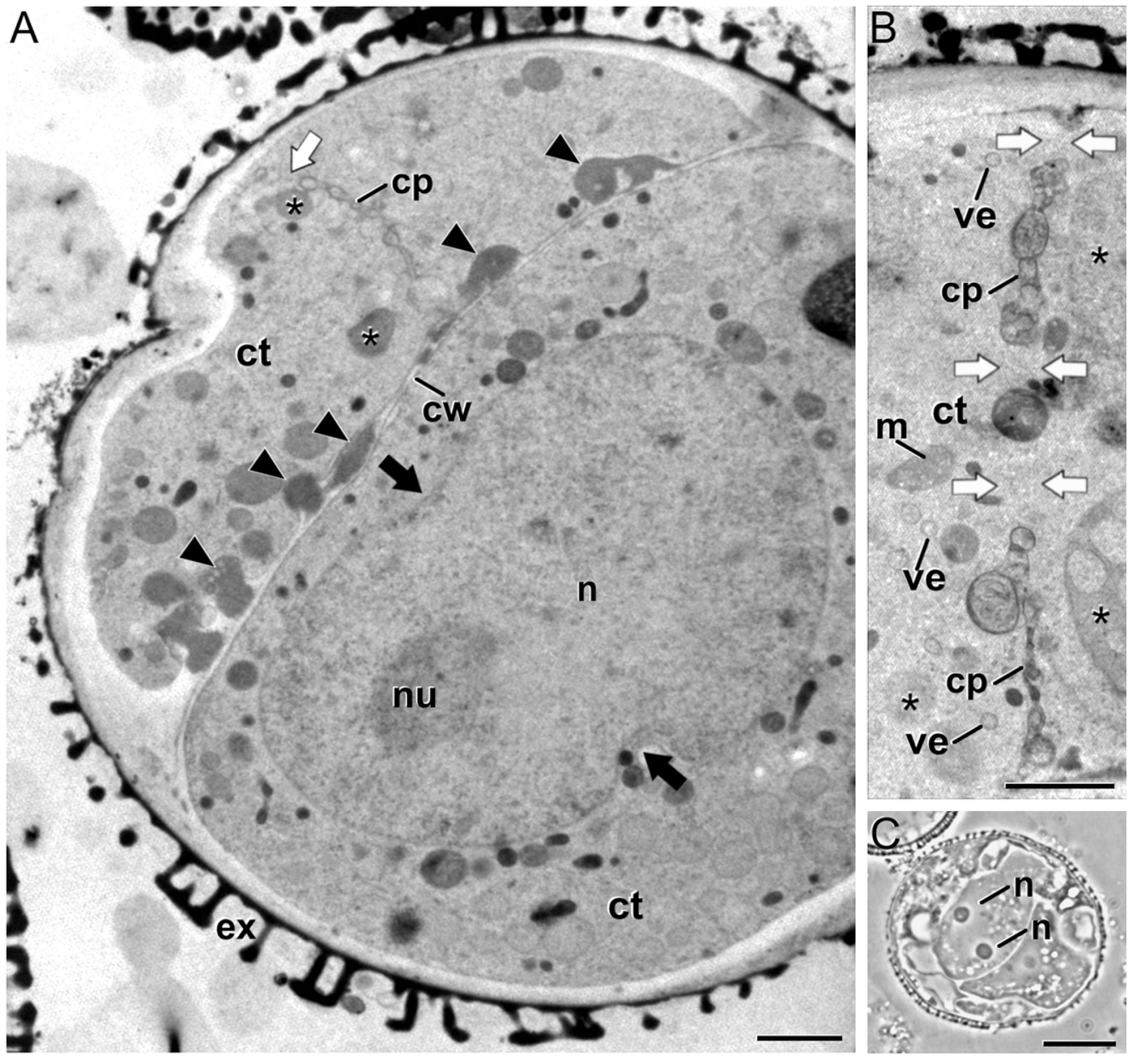
**Embryogenic *B. napus* microspores with abnormal cell plates and signs of nuclear fusion. (A)** Induced microspore with a complete cell wall (cw) with numerous deposits of excreted material (arrowheads), a gapped cell plate (cp), and a peanut-shaped nucleus (n). The black arrows point to nuclear constrictions indicative of a recent nuclear fusion. **(B)** Abnormal, incomplete cell plate with abundant gaps (white arrows) that connect the cytoplasms of the daughter cells, and organelles such as dense vacuolar compartments (asterisks) close to the cell plate. **(C)** Embryogenic microspore with two nuclei coexisting in the same cytoplasm. Ct, cytoplasm; ex, exine; m, mitochondrion; nu, nucleolus; ve, Golgi-derived vesicle. Bars in A: 10 μm; B: 1 μm; C: 200 nm.

Some embryogenic microspores presented continuous cell walls morphologically similar to conventional walls with the only exception of the irregularities and deposits of excreted material (arrowheads in **Figure 2A**) previously described (Corral-Martínez et al., 2013). However, we also observed often dividing cells with abnormal cell plates and cell walls. Abnormal cell plates were characterized by an irregular architecture, with numerous tubular profiles and abundant openings (fenestrae) that permitted the contact between the cytoplasm of daughter cells (white arrows in **Figures 2A,B**). In conventional cell plates, planar fenestrated profiles typically arise at the mid stage of cytokinesis. This stage is defined by the presence of a transitional, ring-shaped phragmoplast (Austin et al., 2005) and the targeted delivery of Golgi-derived vesicles to the growing edges of the cell plate, but not to the maturing central part (Seguí-Simarro et al., 2004). However, in the unusual cell plates of embryogenic microspores such a ring phragmoplast was absent, as revealed by the close proximity of cell organelles and dense vacuoles (Corral-Martínez et al., 2013) to both central and peripheral domains of the cell plate (asterisks in **Figures 2A,B**). In addition, the few Golgi-derived vesicles identified were observed randomly dispersed throughout the tubular cell plate (**Figure 2B**). These data would be indicating that in these cells, the final stage of cytokinesis (the formation of a planar fenestrated sheet) is disturbed. In parallel, we identified embryogenic microspores with binucleated cells (**Figure 2C**) and cells containing nuclei larger than usual, occasionally with a peanut-like morphology (**Figure 2A**), indicative of the recent occurrence of nuclear fusion events (Seguí-Simarro and Nuez, 2007, 2008b; Corral-Martínez et al., 2011).

**FIGURE 3:**
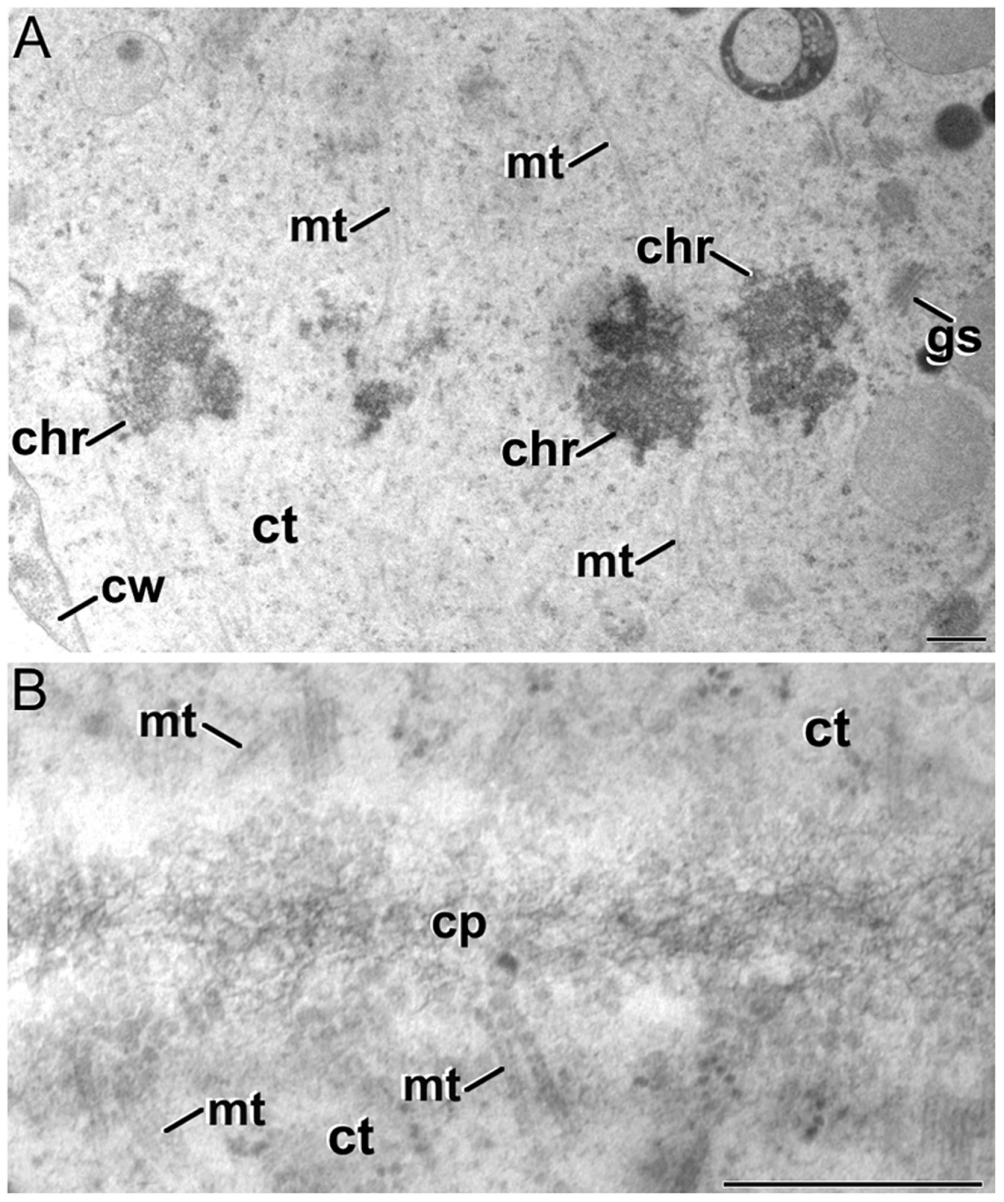
**Dividing cells of *B. napus* embryogenic microspores. (A)** Metaphasic chromosomes (chr) at the cell equator of a dividing cell. **(B)** Tubulo-vesicular network (TVN) cell plate (cp) of a dividing cell at early telophase. ct, cytoplasm; cw, cell wall; gs, Golgi stack; mt, microtubules. Bars: 500 nm.

Thus, we analyzed embryogenic microspores at previous stages of cell division in order to identify the initials of the cytokinesis defects. A detailed observation of mitotic cells did not reveal any structural abnormality with respect to the normal architecture of the mitotic machinery. As seen in **Figure 3A**, metaphasic cells presented the typical condensed chromosomes and an organelle-free region where the abundant parallel microtubules of the mitotic spindle could be identified. Images of cells showing the microtubular scaffold of the mitotic spindle and the solid phragmoplast appeared normal, as well as the cell plate initials that appeared correctly assembled (data not shown). Later on, at early telophase, the TVN cell plate typical from the solid phragmoplast stage was clearly identified together with abundant vesicles and microtubules near the cell plate (**Figure 3B**). Together, these observations pointed to the existence of problems during late stages of cytokinesis, but not before. It seemed that at the ring phragmoplast stage, where cell plate flattening and maturation starts, cytokinesis was somehow blocked and abnormal cell walls were finally formed.

### The Presence of a Thick, Distinct Layer Beneath the Intine is a Differential Feature of Embryogenic Microspores

**FIGURE 4:**
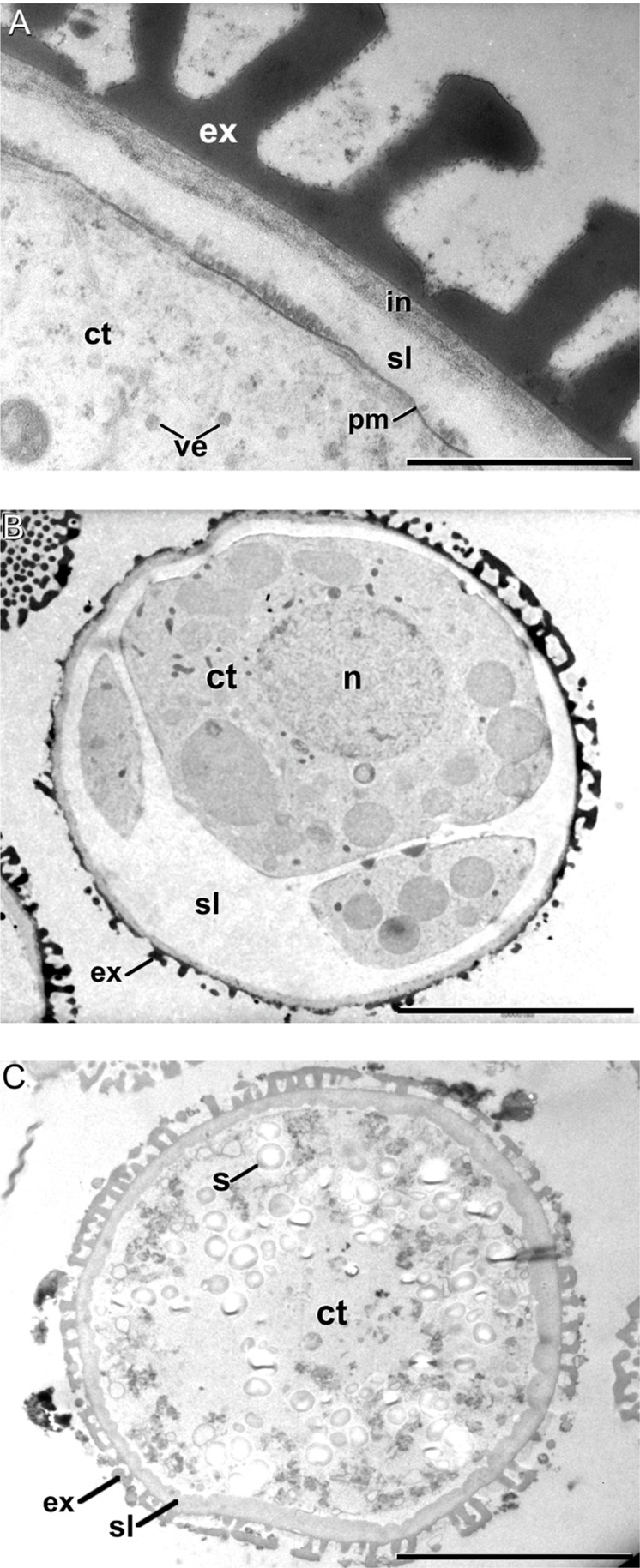
**The subintinal layer of *B. napus* embryogenic microspores. (A)** Electron light subintinal layer (sl) deposited beneath the intine (in) of a embryogenic microspore. **(B)** Overview of an embryogenic microspore showing a continuous subintinal layer. **(C)** Pollen-like structure showing a thickened, uniform and electron dense intine. Ct, cytoplasm; ex, exine; n, nucleus; pm, plasma membrane; s, starch granule; ve, vesicle. Bars in A: 200 nm, B, C: 10 μm.

Soon after induction, we observed that cultured microspores presented an extra layer deposited just between the intine and the plasma membrane (**Figure 4A**). From now on, we will refer to this layer as the *subintinal layer*. The subintinal layer is defined as a continuous layer of irregular thickness, alternating thick and thin domains, with an electron light appearance in osmium-treated, epoxy-embedded samples. Such a layer, including all the mentioned features, was exclusively observed in embryogenic microspores (**Figure 4B**). Pollen-like structures also presented a thickened inner layer (**Figure 4C**). However, this layer was homogeneous in thickness and very similar to the intine in terms of electron density. Indeed, at low magnification the coat of pollen-like structures appeared to be composed by the exine and a thickened intine, in contrast to the three layers of embryogenic microspores (compare **Figures 4B,C**). This suggested an independent nature for the subintinal layer. As expected, this layer was absent from young microspores and pollen grains not subjected to isolation and induction treatments (**Figures 6A,B**, respectively). Therefore, it seemed that the subintinal layer is a structure assembled exclusively in embryogenic microspores.

### Embryogenic Microspores Present Callose-rich Subintinal Layers and Cell Walls

**FIGURE 5:**
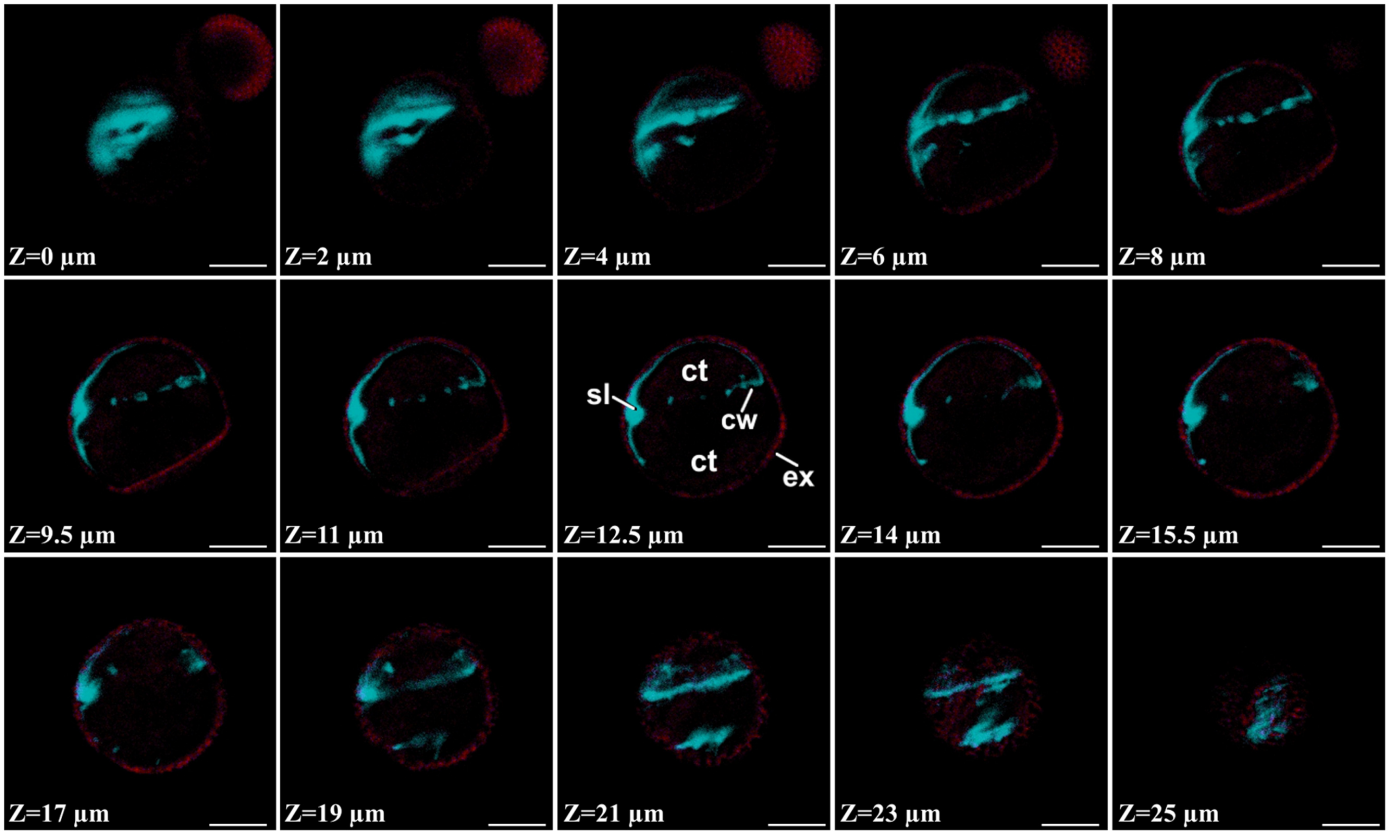
**Aniline blue staining of a 3 day-old embryogenic microspore.** Confocal Z-series covering an entire micropore where the blue signal corresponds to aniline blue staining and the red signal corresponds to exine (ex) autofluorescence. ct, cytoplasm; cw, cell wall; sl, subintinal layer. Bar: 10 μm.

The subintinal layer of embryogenic microspores had in general a texture resembling the callose-rich wall of meiocytes (Supplementary Figures S1A,B), as it can be deduced by comparing **Figure 4B** and Supplementary Figure S1C. For this reason, we investigated the putative callosic nature of this layer by two parallel approaches: staining with aniline blue, a callose-binding fluorescent dye, and immunogold labeling with anti-callose antibodies. Just after the induction treatment, abundant aniline blue fluorescence was found at specific regions throughout the embryogenic microspore. **Figure 5** shows a series of confocal slices from a representative embryogenic microspore, covering different planes from pole to pole. In this series, it is observed that callose accumulated below the exine in a non-uniform manner, alternating wide regions with bright fluorescence and thin regions with faint fluorescence (**Figure 5**). Interestingly, we also found aniline blue staining in the newly formed cell walls separating the daughter cells. Fluorescence was not uniform, indicating a differential callose accumulation at different cell wall domains. In some cell walls, cytoplasmic bridges were found connecting daughter cells through the regions devoid of aniline blue staining (Supplementary Figure S2). This finding, consistent with our observations in TEM micrographs (**Figure 2B**), confirmed the existence of incomplete, callose-rich cell walls. On the contrary, no evident aniline blue fluorescent signal was observed at the thickened coat of pollen-like structures or in the cell walls of small globular MDEs (Supplementary Figures S3A,B). Therefore, we confirmed the unusual and transient accumulation of callose in embryogenic microspores.

**FIGURE 6:**
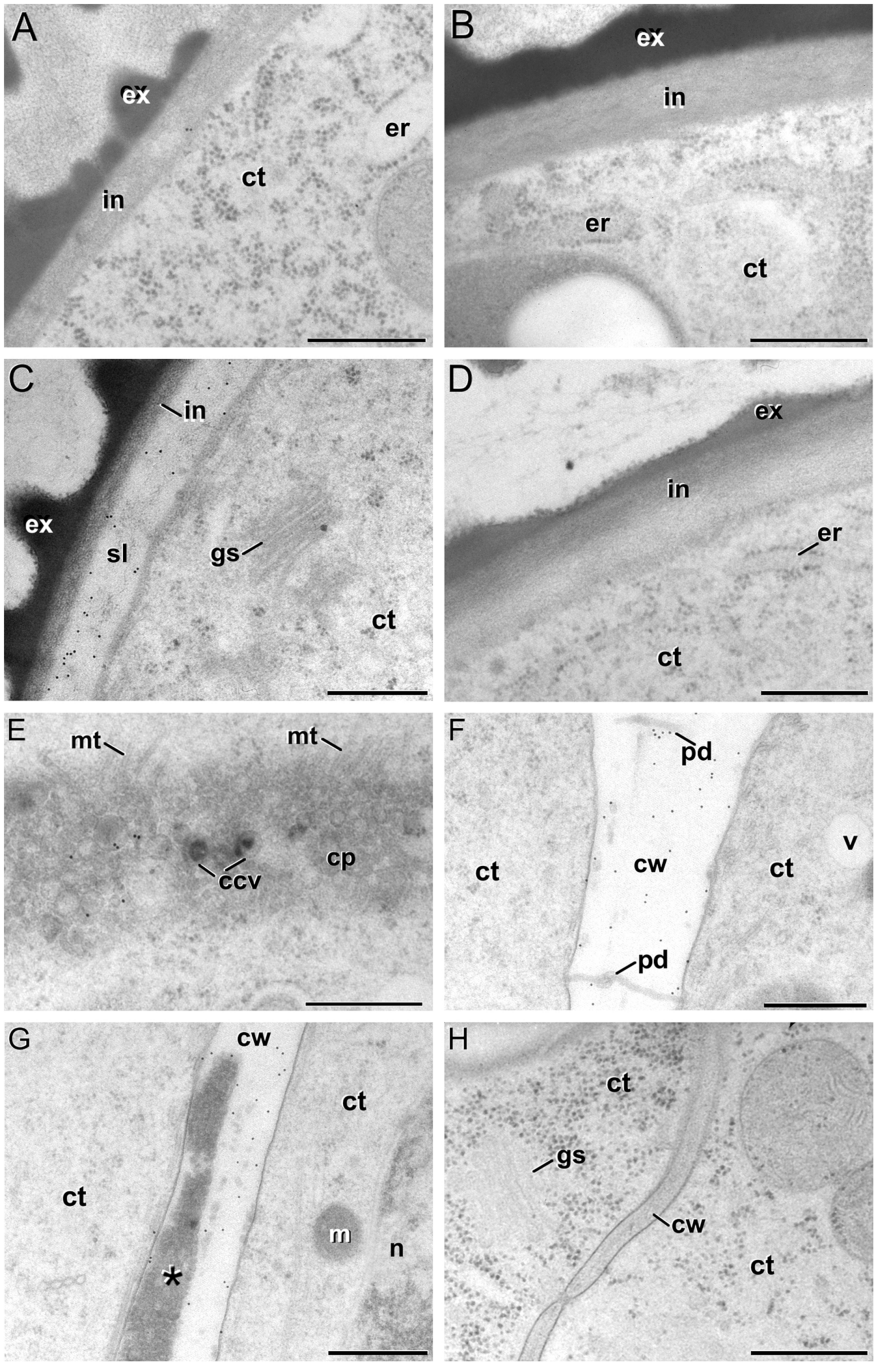
**Anti-callose immunogold labeling. (A,B)** *In vivo* vacuolate microspore **(A)** and pollen grain **(B)**. Note the absence of a subintinal layer in these cell types. **(C)** Embryogenic microspore with abundant gold particles decorating the subintinal layer (sl). **(D)** Pollen-like structure with a thickened intine without anti-callose gold particles. **(E–G)** Developing cell plate **(E)** and mature cell wall **(F,G)** of embryogenic microspore cells, decorated with anti-callose gold particles. Note in **(G)** that callose labeling is absent from the deposits of excreted material (asterisk). **(H)** MDE cell without anti-callose gold particles in the cell wall. Ccv, clathrin-coated vesicle; ct, cytoplasm; cp, cell plate; cw, cell wall; er, endoplasmic reticulum; ex, exine; gs, golgi stack; in, intine; m, mitochondria; mt, microtubules; n, nucleus; pd, plasmodesma; v, vacuole. Bars: 500 nm.

Then, we performed a more comprehensive study at different stages before induction (during *in vivo* microspore development), just after induction, and 10 days after induction (in MDEs) by means of immunogold labeling with anti-callose antibodies. Positive controls with tomato meiocytes (Supplementary Figure S1D) showed an intense labeling of the callosic walls. Negative controls excluding the secondary antibody provided no labeling at all (Supplementary Figures S1E,F). Vacuolate microspores, during their *in vivo* development within the anther, showed almost no callose at their coat (**Figure 6A**). The exine was completely devoid of labeling and only clusters of 2–3 gold particles could be rarely observed at the intine, usually close to the plasma membrane. This was consistent with the thin callose layer described as necessary for exine formation and pollen viability (Dong et al., 2005). Pollen grains maturing within the anther showed no labeling at any layer of the pollen coat (**Figure 6B**) except for mature pollen, where anti-callose labeling was found below the pollen apertures (data not shown). After induction, the few cells enclosed within the embryogenic microspores presented a specific anti-callose labeling restricted to the subintinal layer (**Figure 6C**). In contrast, the thickened inner layer of pollen-like structures showed no labeling (**Figure 6D**). This different labeling pattern between embryogenic and pollen-like structures was consistent with their differences at the ultrastructural level. In addition to their different thickness and electron density, these layers also differed in callose contents.

Anti-callose antibodies also decorated the new cell walls created after the first embryogenic divisions. Young, developing cell plates (**Figure 6E**) but also older, mature cell walls (**Figure 6F**) showed immunogold labeling dispersed throughout the wall. Callose is a common component of developing somatic-type cell plates, but is progressively replaced by cellulose during the maturation stages (Samuels et al., 1995). With the exception of plasmodesmata, callose is absent from mature cell walls. Therefore, its persistence should be considered an abnormal feature of embryogenic microspores. In walls with deposits of excreted material (Corral-Martínez et al., 2013), callose was always absent from these deposits (**Figure 6G**). This suggested that callose deposition and secretion of cytoplasmic material were independent processes. In older stages of microspore embryogenesis, the cell walls of suspensor-bearing MDEs did not show decoration with anti-callose antibodies (**Figure 6H**), as expected for mature cell walls derived from somatic-type cytokinesis. In other words, the unusual presence of callose in mature cell walls of few-celled embryogenic microspores appeared to be a transient phenomenon, associated to the first stages of this developmental switch, and absent from MDEs with a clearly established embryogenic pattern.

### Cellulose is Absent in the Callose-Rich Cell Walls of Just Induced Embryogenic Microspores

Due to the abnormal pattern of callose deposition observed in embryogenic cells, we next studied whether this might have an influence on its subsequent replacement by cellulose. For this, we stained microspore cultures with two cellulose-specific stains: calcofluor white, conventionally used to stain cellulose, and Pontamine Fast Scarlet 4B (S4B), described as more specific for cellulose than calcofluor white (Thomas et al., 2013). Calcofluor white staining revealed a noticeable and continuous cellulose signal at the intine of pollen-like structures, being more intense at the regions just below the apertures (Supplementary Figure S4A). In contrast, the induced microspores and MDEs exhibited variable patterns of cellulose deposition (Supplementary Figures S4B,C). To further investigate this unusual observation and the possible interaction between cellulose and callose, we performed a double staining of just induced microspore culture samples with aniline blue and S4B. We analyzed 289 cultured microspores from different randomly chosen microscope fields. From this microspore population, 217 (75.1%) showed no staining, only exine autofluorescence. Most likely, these microspores would be either dead or arrested in development, as usual in microspore cultures of all inducible species (Seguí-Simarro and Nuez, 2008a). The remaining 72 microspores (24.9%) showed aniline blue staining, S4B staining, or both. We carefully analyzed these 72 microspores and categorized them into four groups according to their morphology, internal architecture and double staining pattern.

**FIGURE 7:**
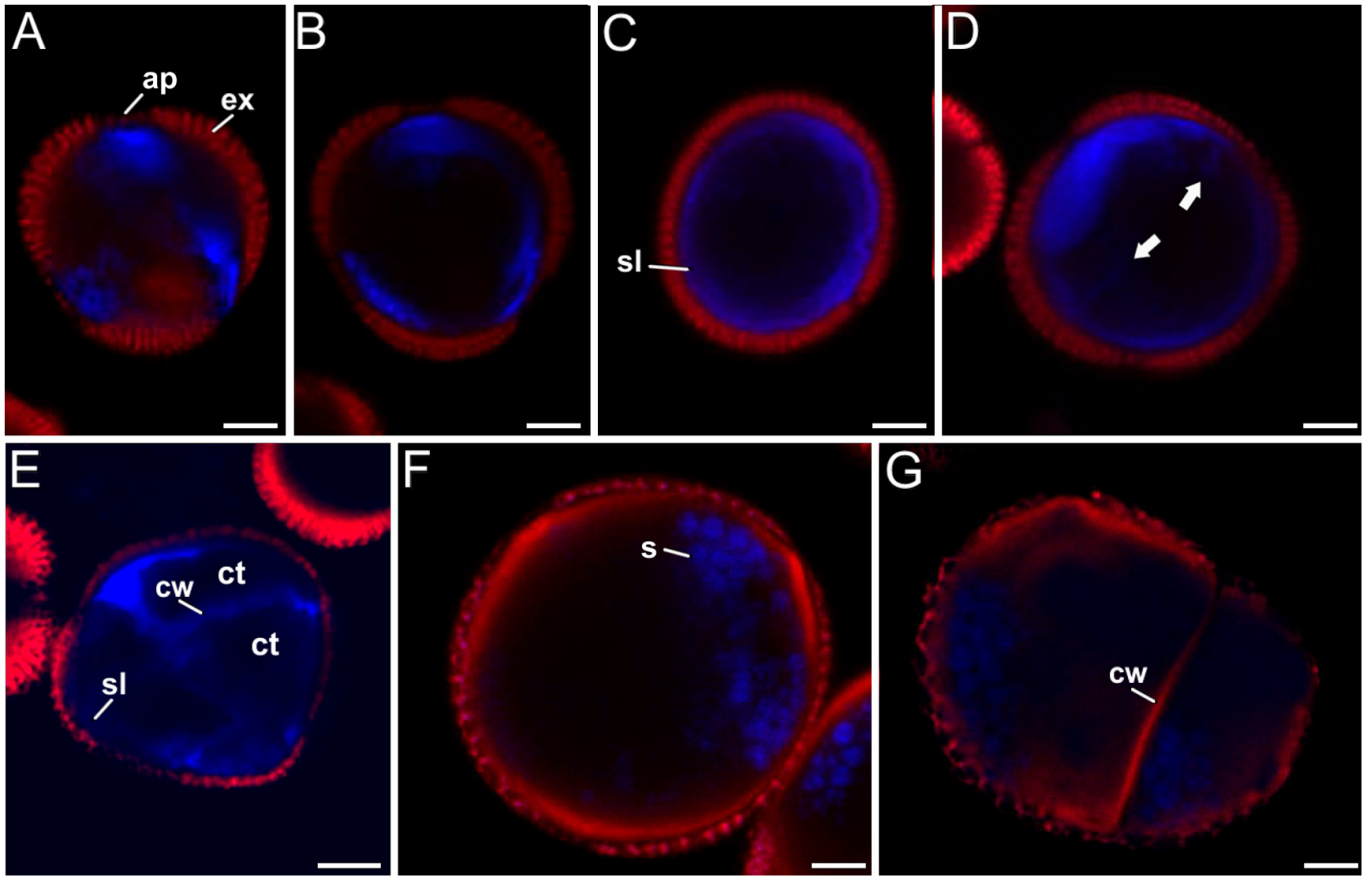
**Callose (blue) and cellulose (red) double staining with aniline blue and S4B. (A–D)** Unicellular (not yet divided) microspores developing a callose-rich subintinal layer (group 1). **(E)** Three-celled microspore (group 2) with callose-rich subintinal layer (sl) and inner cell walls (cw). Note the absence of red cellulose signal. **(F)** Pollen-like structure (group 3) showing cellulose signal (red) below the exine, but not blue callose signal. **(G)** Microspore with unusual patterns of development (group 4) showing cellulose signal (red) below the exine and in the inner cell wall. The blue spots in **(F–G)** correspond to unspecific staining of starch granules by aniline blue. The dark red signal corresponds to exine (ex) autofluorescence. Bars: 5 μm.

- *Group 1*: 12.5% of the microspores presented a slightly lobulated shape and a size similar to vacuolate microspores (**Figures 7A–D**). No embryogenic divisions were identified in their cytoplasm. No S4B staining was observed in any part of any of these cells. However, intense aniline blue fluorescence was consistently found at the region below the apertures (**Figure 7A**), in some cases extended beyond these regions (**Figure 7B**), and even along the entire subintinal layer (**Figure 7C**). Some of them also presented small stubs penetrating into the cytoplasm (arrows in **Figure 7D**), although a clear cell wall could not be observed in phase contrast images. Together, these microspores with increasing levels of aniline blue staining at their periphery were suggestive of different stages in the formation of the subintinal layer, prior to the first embryogenic division. Such formation would start at the region of the apertures, and would extend centrifugally to eventually cover the whole microspore.

- *Group 2*: 40.3% of the microspores presented the typical features of embryogenic microspores. In addition to the morphological features mentioned above for the first group, we clearly observed in all of them continuous (but variable in thickness) subintinal layers and cell walls, positive for aniline blue staining but negative for S4B staining (**Figure 7E**).

- *Group 3*: 43.1% of the microspores clearly showed pollen-like features, such as enlarged size, oval shape, dense cytoplasm, absence of symmetric divisions, and abundance of starch granules. When observing the S4B staining, these cells revealed another common feature: all of them but one presented a bright and continuous staining at the thickened intine layer (**Figure 7F**). This trait was remarkably different from the embryogenic microspores, devoid of cellulose at any cell wall region. Some of the cellulose-containing, pollen-like structures (15.3% of the total) were also positive for aniline blue staining at the regions below the apertures (data not shown), and only one (1.4% of the total) was positive only for aniline blue. In some double stained pollen-like structures, starch granules seemed clearly delineated by aniline blue staining (**Figure 7F**). To verify this, we isolated starch rich, mature pollen grains from anthers and stained them with aniline blue, confirming that pollen starch granules were also stained by aniline blue (Supplementary Figure S5).

- *Group 4*: 4.2% of the microspores presented abnormal patterns of development, combining features of pollen-like structures such as starch grains and large sizes with features of embryogenic microspores, such as cell divisions and new cell walls (**Figure 7G**). In addition, these cells presented high levels of cytoplasmic vacuolation, exine rupture at different regions of their surface, and asymmetric division patterns. These structures have been previously described as non-embryogenic, arresting, and dying after one or few division rounds (Corral-Martínez et al., 2013). Two microspores of this group (2.8% of the total) showed only cellulose staining, which delineated both the inner cell walls and the thickened intine (**Figure 7G**), and only one (1.4% of the total) presented both, cellulose and callose signal, mostly at the regions below the apertures.

As for MDEs, in young 4–8 MDEs, cellulose deposition was very scarcely found in the outer walls, being absent from the inner walls (data not shown). Equivalent (quadrant and octant) suspensor-bearing MDEs presented a similar cellulose pattern at the embryo proper domain, whereas suspensor cell walls were clearly delineated by cellulose staining (Supplementary Figure S4B). This pattern, however, changed from globular MDEs onward (Supplementary Figure S4C), where staining consistently decorated all the cell walls of the embryo proper in a pattern similar to a conventional mature cell wall. Together, these results indicated that the first embryogenic divisions of the induced microspore are defined by abnormal patterns of callose and cellulose deposition, establishing a clear correlation between the morphological features that define a microspore as embryogenic, the presence of callose at both the subintinal layer and the cell walls, and the total absence of cellulose. This pattern, however, changes in MDEs, where cellulose is progressively more present in the cell walls of developing MDEs, being abundant at the stages of globular and beyond, as expected for normal somatic-type cell walls.

### Altered Ca^2+^ Levels Modify the Patterns of Callose and Cellulose Deposition in the New Cell Walls of Embryogenic Microspores

Once demonstrated that the abundance of callose in the newly formed cell walls is a feature inherent to the developmental switch toward embryogenesis, we wanted to find the cause of such abundance. Due to the role assigned to Ca^2+^ in the regulation of callose deposition and in the inhibition of cellulose synthesis at the cell plate (Hayashi et al., 1987; Verma and Hong, 2001), we applied caffeine and BA to microspore cultures, and observed their effects in embryogenic microspores. Caffeine is known to redistribute the intracellular Ca^2+^ by altering transmembrane calcium gradients, thereby reducing the levels of Ca^2+^ available for callose synthases at the cell plate (Samuels and Staehelin, 1996). As a consequence, Ca^2+^-deprived callose synthases cannot synthesize callose at normal levels (Kakimoto and Shibaoka, 1992). In turn, BA is a membrane fluidizer that induces major but transient elevations of cytosolic Ca^2+^ (Saidi et al., 2009).

**FIGURE 8:**
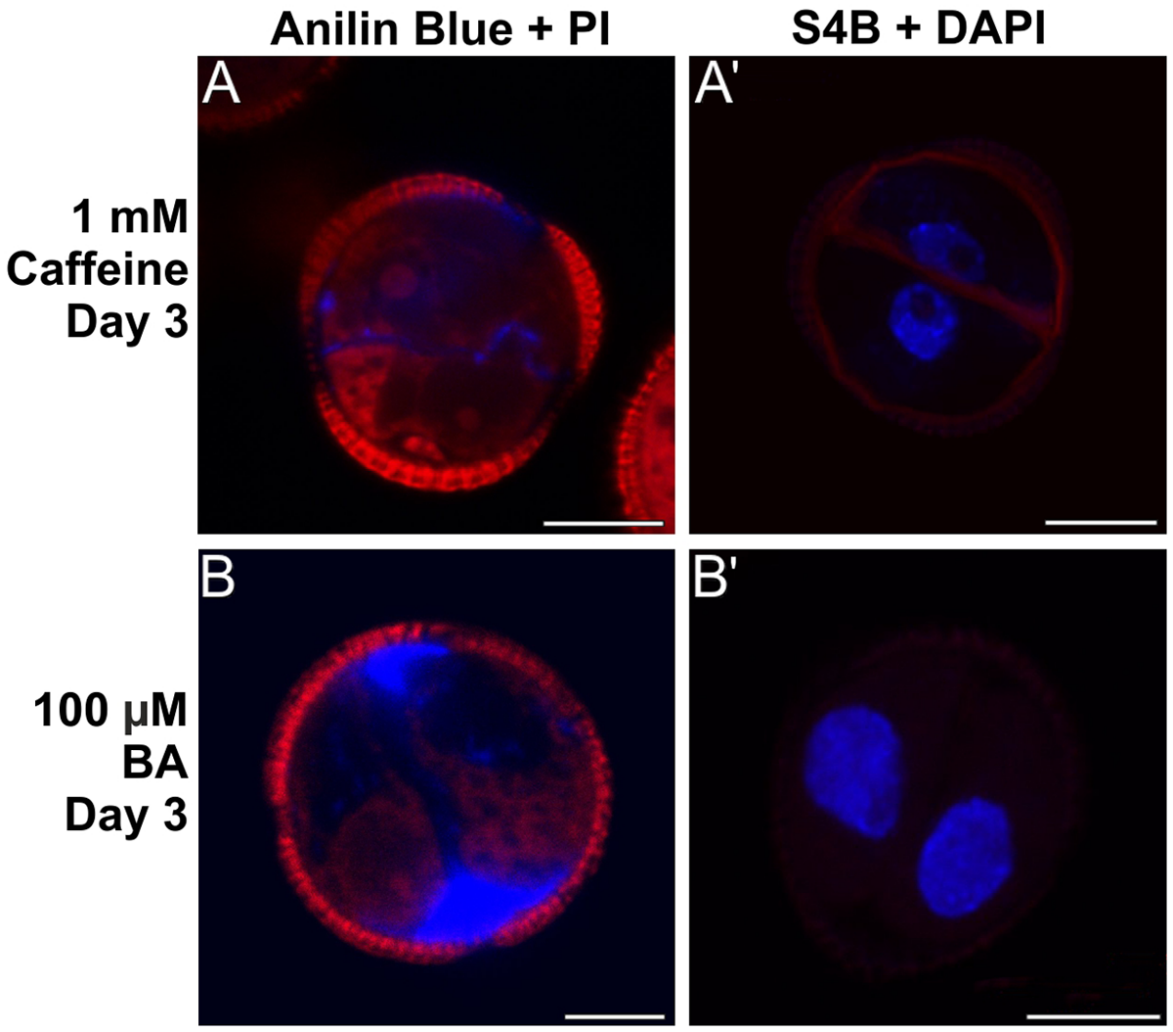
**Effect of caffeine and benzyl alcohol (BA) on callose and cellulose deposition. (A,A’)** Three day-old embryogenic microspores cultured with 1 mM caffeine and stained with aniline blue + PI **(A)** and S4B + DAPI **(A’)**. **(B,B’)** Three day-old embryogenic microspores cultured with 100 μM BA and stained with aniline blue + PI **(B)** and S4B + DAPI **(B’)**. Bars: 10 μm.

When 1 mM caffeine was added to microspore cultures, callose-containing cell walls were observed between daughter cells, but they were thinner and aniline blue staining was less intense and extense (**Figure 8A**) than in control cells. The subintinal layer was also thinner, discontinuous and irregularly stained by aniline blue. Interestingly, S4B staining revealed the continuous presence of cellulose along the cell walls and subintinal layer of ∼50% of the cells (**Figure 8A’**). As opposed to control cultures, 21 day-old caffeine-treated cultures did not produce MDEs. In contrast, 100 μM BA-mediated increase of intracellular Ca^2+^ levels produced a increase in callose deposition, principally in the subintinal layer as revealed by the abundant aniline blue staining (**Figure 8B**). S4B staining, however, evidenced the absence of cellulose in these cells (**Figure 8B’**). Interestingly, 21 days after induction, BA-treated cultures produced ∼600 MDEs/ml (Supplementary Figure S6), which represented 240% of the yield of control cultures (∼250 MDEs/ml). Together, these results indicated that alteration of Ca^2+^ levels by caffeine and BA affected the amount of callose deposited in the cell walls of embryogenic microspores. Caffeine-mediated Ca^2+^ redistribution reduced callose deposition and allowed for the synthesis of cellulosic (conventional) walls in some microspores, which suggested that the lack of cellulose was due to a Ca^2+^-mediated massive deposition of callose. On the other hand, BA-mediated increase of intracellular Ca^2+^ produced more callose in the subintinal layer and, as expected, cellulose-free cell walls. In addition to this, it seemed that the addition of BA was markedly beneficial for microspore embryogenesis, since 2.4x more MDEs were produced.

## Discussion

It is clear from this and other works (Solis et al., 2008; Barany et al., 2010; Dubas et al., 2013) that the cell wall is a highly dynamic structure whose architecture, arrangement, and composition changes dramatically as the microspore enters embryogenesis and then transforms into a MDE. We have demonstrated that a direct consequence of the exposure of microspores to the inductive treatment is the production of unusual walls with a unique composition. The microspore intine, described to have a pectocellulosic nature (Sitte, 1953), retained a cellulose-rich composition in pollen-like structures, as previously described for pollen (Heslop-Harrison, 1979). In contrast, the subintinal layer presented a dramatically different composition, indicating that it is not an extension of the intine, but a different structure originated *de novo* upon induction. Interestingly, the two cell walls created *de novo* in embryogenic microspores (inner cell walls and the subintinal layer) had the same appearance and composition, which was different from previously existing walls (the intine), but also from the walls of MDEs. It seems that during the first stages of the switch, the mechanisms of synthesis and/or deposition of cell wall components are altered, giving rise to the transient formation of atypical walls. The origins, implications and consequences of the presence of these walls are discussed next.

### The Subintinal Layer is an Early Marker of the Androgenic Switch

The presence of additional layers beneath the coat of embryogenic microspores in different species has been previously reported in the literature, but only occasionally (Rashid et al., 1982; Zaki and Dickinson, 1990; González-Melendi et al., 1995; Solis et al., 2008; Dubas et al., 2013). Perhaps, the reason why this layer has not been studied more in detail relays on the fact that TEM images of chemically fixed embryogenic microspores frequently showed cell retraction and waviness of plasma membrane (Telmer et al., 1993; González-Melendi et al., 1995; Maraschin et al., 2005; Seguí-Simarro et al., 2006), making it difficult to figure out whether the electron light spaces observed are real structures or just retraction artifacts. In addition, it is known that aldehyde fixation may induce callose deposition (Hughes and Gunning, 1980), which may raise doubts about the presence of callose in chemically fixed embryogenic microspores. We demonstrated that the callose-rich subintinal layer of embryogenic microspores is a real entity, not induced by chemical fixation. Thus, it is pertinent to ask whether this layer is a cause or a consequence of the developmental switch.

In plant development, the synthesis of a callose-rich layer is generally related to the developmental fate of the cells that synthesize it. The self-aggregating properties of the helical callose molecules, and the dense, insoluble nature of the highly hydrated but semipermeable callose gels make callose deposits ideal for creating physical and chemical barriers (Albersheim et al., 2011). Indeed, during meiosis callosic walls isolate the developing microspore and megaspore mother cells, acting as a physical barrier against premature swelling and bursting (Zhang et al., 2002), and also as a ‘molecular filter’ to allow for the expression of their specific developmental programs without the interference of the surrounding diploid tissues (Heslop-Harrison and Mackenzie, 1967; Abramova et al., 2003). Aside of natural processes, callose has been observed in internodal cells of *Humulus lupulus* committed to organogenesis (Fortes et al., 2002), and in cells of *Trifolium* (Maheswaran and Williams, 1985) and *Cichorium* (Dubois et al., 1991) reprogrammed to somatic embryogenesis. The deposition of a callose layer covering *in vitro*-induced young globular embryos was reported during somatic embryogenesis in *Camellia japonica* leaves (Pedroso and Pais, 1992). Most importantly, it was found that when zygotic embryos of *Eleutherococcus senticosus* are exposed to osmotic stressors such as mannitol or sucrose, single epidermal cells develop a surrounding callose layer between the plasma membrane and the cell walls, similar to the subintinal layer we hereby describe, and then enter somatic embryogenesis (You et al., 2006). These authors stated that plasmolysis-induced callose would interrupt cell-to-cell communication, which in turn would stimulate the embryogenic reprogramming. In line with these findings, we observed that all the embryogenic microspores developed a callosic subintinal layer, whereas those non-embryogenic, did not develop it. Since both structures were exposed to the inductive heat shock and only the embryogenic developed an additional subintinal layer, we deduce that this layer would not be a direct consequence of the heat shock. It could also be argued that this layer could be related to the callose layer that appears beneath the intine in germinating pollen grains (Ferguson et al., 1998). However, we never found such a layer in the non-embryogenic, pollen-like structures, which accounts against this possibility. Instead, we propose that the subintinal layer is related to the change in developmental fate toward embryogenesis. We even found some microspores that developed the callosic layer even before the first clear evidence of inner cell walls was found in them (**Figures 7A–D**). Thus, we propose that the callosic subintinal layer would be formed in microspores prior to the first embryogenic division, and this would isolate them from the outer environment (the liquid culture media or the cultured anther tissue), creating a suitable environment to start a different developmental program, the embryogenic program. It can also be concluded that the deposition of a callose-rich layer, as a step previous to *in vitro* embryo development, would be a common marker associated to reprogrammed cells during different types of *in vitro* morphogenesis, including somatic and microspore embryogenesis.

### The Abnormal Inner Walls of Embryogenic Microspores are Due to Disrupted Cytokinesis

We showed that the cell walls of embryogenic microspores are abnormal in terms of both structure and composition. For us, the most reasonable cause for this is that cytokinesis proceeds normally up to the fenestrated sheet-transitional phragmoplast stage of cytokinesis, and then it is disrupted. Our results are consistent with this hypothesis, since we did not observe any structural alteration up to this stage. Once the cell plate is established at the cell equator, callose synthesis is the driving force that transforms the architecture of the developing cell plate. Callose is an amorphous polymer proposed to provide the fluidity needed to rapidly develop an initial membranous scaffold (Samuels et al., 1995) and to respond to the guidance mechanisms that insert the cell plate at specific sites of the parental wall (Xie et al., 2012). Newly synthesized callose widens the cell plate membranous tubules, as it spreads over the membrane inner surface (Samuels et al., 1995). At the end of this spreading phase, somatic-type cell plates resemble irregular sheets with numerous openings (Samuels et al., 1995; Seguí-Simarro et al., 2004, 2008). This is how the cell walls look like in our micrographs of abnormally walled *B. napus* embryogenic microspores (**Figures 2A,B**). Once the spreading phase is accomplished in somatic-type cytokinesis, the transition from a fluid and wrinkled immature cell plate to a stiff and straight cell wall is associated to deposition of cellulose and pectins and the parallel removal of callose (Kakimoto and Shibaoka, 1992; Samuels et al., 1995; Drakakaki, 2015). However, we showed that callose is not replaced by cellulose in the gapped and incomplete walls of embryogenic microspores. Callose does not form crystalline associations with adjacent strands, as cellulose does (Samuels et al., 1995). Thus, a callose-rich cell wall would in principle be more fluid than a conventional, cellulose-rich cell wall with no callose. In addition, β-1,3-glucans such as callose tend to gelate. It was suggested that the formation of hydrogels by callose would allow insertion or co-gelation with other polymers in the cell plate (Brown and Lemmon, 2009). It is also known that callose gelation can be increased upon heating (Stone and Clarke, 1992). Conceivably, the combination of a mild heat stress, callose accumulation and reduced cellulose contents would confer the newly formed cell walls of embryogenic microspores new properties, different from those of conventional walls. These walls would be more difficult to flatten and close through the conventional mechanisms of cell plate maturation, giving rise to irregular final walls, wrinkled and incomplete, as those shown in **Figures 2A,B**.

All this considered, we propose that the very first division rounds in embryogenic microspores are impaired at the stage of callose replacement by cellulose and as a consequence, the cell walls produced are defective. In support of this, similar phenotypes of abnormal cell plates linked to callose abundance and/or scarce or absent cellulose have also been reported in other experimental systems. For example, in the *Arabidopsis* cytokinesis-defective *cyt1* mutant, the persistence of callose affected cell plate maturation, preventing cellulose synthesis and producing incomplete cell walls (Nickle and Meinke, 1998). Onion root cells treated with the herbicide dichlobenil, that prevents cellulose synthesis, showed reticulate and wavy cell plates with dramatically increased levels of callose (Vaughn et al., 1996). Furthermore, the *Arabidopsis rsw1* null mutant, where the activity of the RSW1/CeSA1 cellulose synthase gene is knocked down, produced thin, highly undulated, and frequently interrupted primary cell walls (Beeckman et al., 2002).

### Increased Ca^2+^ Levels may be Responsible for Prolonged Callose Synthase Activity and Cellulose Inhibition

According to our results, it is pertinent to ask why callose persists and cellulose is not deposited in the first cell walls formed in embryogenic microspores. One candidate to mediate in this process is Ca^2+^ signaling, a highly conserved mechanism for temperature sensing among land plants (Horvath et al., 2012). Heat stress, among other treatments, is known to alter the properties of plant cell plasma membranes (reviewed in Horvath et al., 2012). Heat shock tends to fluidize plasma membranes, becoming leaky to ions and thus favoring uncontrolled influxes of Ca^2+^ and rapid, transient and proportional elevations of cytosolic Ca^2+^ in moss cells (Saidi et al., 2009), higher plants (Gong et al., 1998), and in *B. napus* embryogenic microspores (Pauls et al., 2006). In addition, high levels of intracellular Ca^2+^ are required for the activity of cell plate-specific, Ca^2+^-dependent callose synthases (reviewed in Verma, 2001; Drakakaki, 2015). Related to this, our experiments with caffeine and BA suggested a possible relationship between the intracellular Ca^2+^ levels and the amount of callose deposited, which shows that the synthases involved in the development of the new cell wall and the subintinal layer are Ca^2+^-sensitive. In somatic-type cell walls, high Ca^2+^ levels are known to cause the shift toward callose production, whereas Ca^2+^ levels must be reduced for cellulose synthesis to initiate during cell plate maturation. Indeed, prolonged high Ca^2+^ levels may extend callose synthesis in time and inhibit the shift from callose to cellulose synthesis (Verma, 2001). Our hypothesis is that a similar scenario might be occurring during the formation of the first cell walls in *B. napus* embryogenic microspores. Our experiments showed that caffeine decreased the presence of callose in both the subintinal layer and the inner cell walls, probably due to a reduction of the Ca^2+^ available for callose synthases. As a consequence, cellulose could be produced. In turn, the use of BA produced even more callose in the subintinal layer, probably due to an increase in the Ca^2+^ levels. In summary, we postulate that heat shock would alter the plasma membrane properties to allow for a Ca^2+^ influx that, among other effects (alteration of phragmoplast integrity, for example), would keep callose synthases active and, at the same time, would inhibit cellulose synthases. In this context, it is interesting to note that the effect of BA added to the effect of heat shock not only in terms of Ca^2+^ influx and callose synthesis, but also of MDE yield. Indeed, several lines of evidence point to the redundancies of the cellular responses triggered by temperature-induced Ca^2+^ signaling and BA-mediated Ca^2+^ influx (Sangwan et al., 2001; Saidi et al., 2009; Horvath et al., 2012). Thus, it is tempting to speculate that BA may enhance or replace the effect of heat shock in cell wall remodeling and perhaps, in the activation of the whole embryogenic program in *B. napus* microspores.

### Callose-rich and Cellulose-deficient Walls may Promote Genome Doubling

Cytokinetic defects typically induce the eventual formation of polyploid cells. Incomplete cell walls with large holes allow for the contact between the nuclei of daughter cells. Even those with smaller gaps may end up producing larger holes, since they are prone to break and collapse into pieces. These abnormal walls are known to facilitate the coexistence of nuclei of different cells in the same cytoplasm (Sunderland and Dunwell, 1974) and their eventual fusion, as demonstrated to occur in many other *in vitro* culture systems and cytokinesis-defective mutants (Mayer et al., 1999; Kasha et al., 2001; Muller et al., 2002; Strompen et al., 2002; Testillano et al., 2004; González-Melendi et al., 2005; Seguí-Simarro and Nuez, 2007; Corral-Martínez et al., 2011; De Storme et al., 2013). Our results showed that the instability and fluidity of callose-rich and cellulose-deficient cell walls, together with the severe cell wall deformations produced by the accumulation of excreted cytoplasmic material (Corral-Martínez et al., 2013), are behind the occurrence of incomplete, broken, or fenestrated cell walls. Hence in some embryogenic microspores, impairment of cytokinesis mediated by excessive callose deposition would generate the structural context necessary to facilitate nuclear fusion and therefore genome duplication, a necessary step widely accepted to produce DH cells, embryos and plants. On the other hand, cells where callose deposition is not so persistent would develop normal walls, which, according to this hypothesis, would preclude nuclear fusion during these initial stages, giving rise to haploid embryos. In turn, it is reasonable to assume that there will also be few embryogenic microspores whose initial phase of defective cytokinesis would last longer, being their cells more prone to undergo more than one round of nuclear fusions. It may also be possible that in a single embryogenic microspore, normally dividing cells coexist with others undergoing defective cytokinesis, giving rise to mixoploid and/or polyploid MDEs, known to occur in *B. napus* (Abdollahi et al., 2012) as well as in other species (Sato et al., 2005).

## Conclusion

**FIGURE 9:**
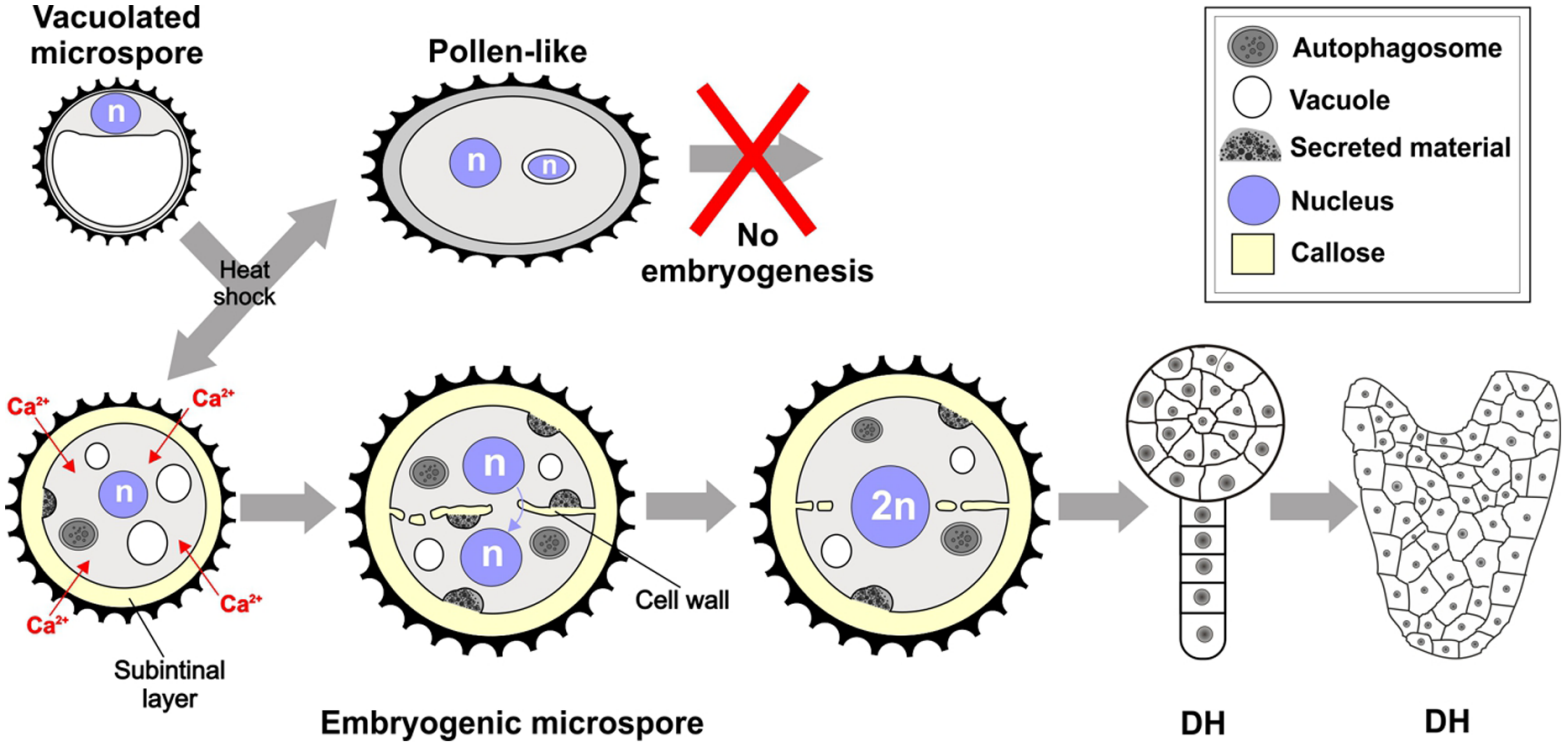
**Formation of the callose-rich subintinal layer and abnormal cell walls upon heat shock induction of embryogenesis in *B. napus* microspores.** See text for further details.

We showed in this work that one of the first signs of embryogenic commitment is the formation of callose-rich, cellulose-deficient subintinal layers and irregular, gapped, and incomplete cell walls (**Figure 9**), both of them absent in pollen-like structures. This is due to the combination of three factors: the formation of large deposits of excreted cytoplasmic material described in Corral-Martínez et al. (2013), the massive deposition of callose, and the inhibition of cellulose synthesis. Callose would be produced by Ca^2+^-dependent callose synthases activated by a raise in intracellular Ca^2+^ levels, which is a direct consequence of the increased permeability of the plasma membrane as a result of the inductive heat shock. Few days after the heat shock, abnormal cell walls are no longer produced, and MDEs present conventional, somatic-type cell walls.

These findings provide new insights that contribute to understand this intriguing, yet largely unknown developmental switch. In turn, our results pose new, open questions. One of them is why pollen-like structures, exposed to the same physico-chemical environment, do not produce callose-rich cell walls. A possible explanation is that these structures may have irreversibly entered the gametophytic-like pathway before the application of the inductive treatment, and this may somehow make them insensitive to the effects of heat shock exposure in terms of induction of embryogenesis. Another question is how and when the cell wall-associated phenomena we described cease, and conventional cell walls are formed in MDEs. It is possible that once the cells return to normal conditions, newly formed conventional walls coexist with the few preexisting callose-rich walls, as we have observed in some globular MDEs. After 1000s of conventional cytokinesis, the relevance of callosic walls would become negligible. However, we cannot rule out the possibility of callose being eliminated after the heat shock by specific 1,3-β glucanases. In favor of this hypothesis, Borderies et al. (2004) correlated the development of maize MDEs with the secretion of several proteins to the culture medium, one of them being a 1,3-β glucanase. It would be interesting to know whether *B. napus* MDEs produce 1,3-β glucanases as well. Finally, it would also be interesting to know whether the process we hereby describe or similar phenomena are triggered in other species where microspore embryogenesis is induced by other stresses different from temperature changes.

## Author Contributions

JMSS designed the research; VPV, PCM, ARS, and JMSS obtained and processed the samples. VPV, ARS, and PCM performed the experiments. JMSS wrote the manuscript.

## Conflict of Interest Statement

The authors declare that the research was conducted in the absence of any commercial or financial relationships that could be construed as a potential conflict of interest.
