# Supplementary material for: Induction of Embryogenesis in Brassica Napus Microspores Produces a Callosic Subintinal Layer and Abnormal Cell Walls with Altered Levels of Callose and Cellulose
Source: Front Plant Sci. 2015 Nov 25;6:1018. doi: 10.3389/fpls.2015.01018 (PMC4658426; doi:10.3389/fpls.2015.01018)
Supplement: Supplementary file 1 [file Data_Sheet_1.PDF]

Induction of embryogenesis in *Brassica napus* microspores produces a callosic subintinal layer and abnormal cell walls with altered levels of callose and cellulose

Verónica Parra-Vega, Patricia Corral-Martínez, Alba Rivas-Sendra and Jose M. Seguí-Simarro

SUPPLEMENTARY FIGURES

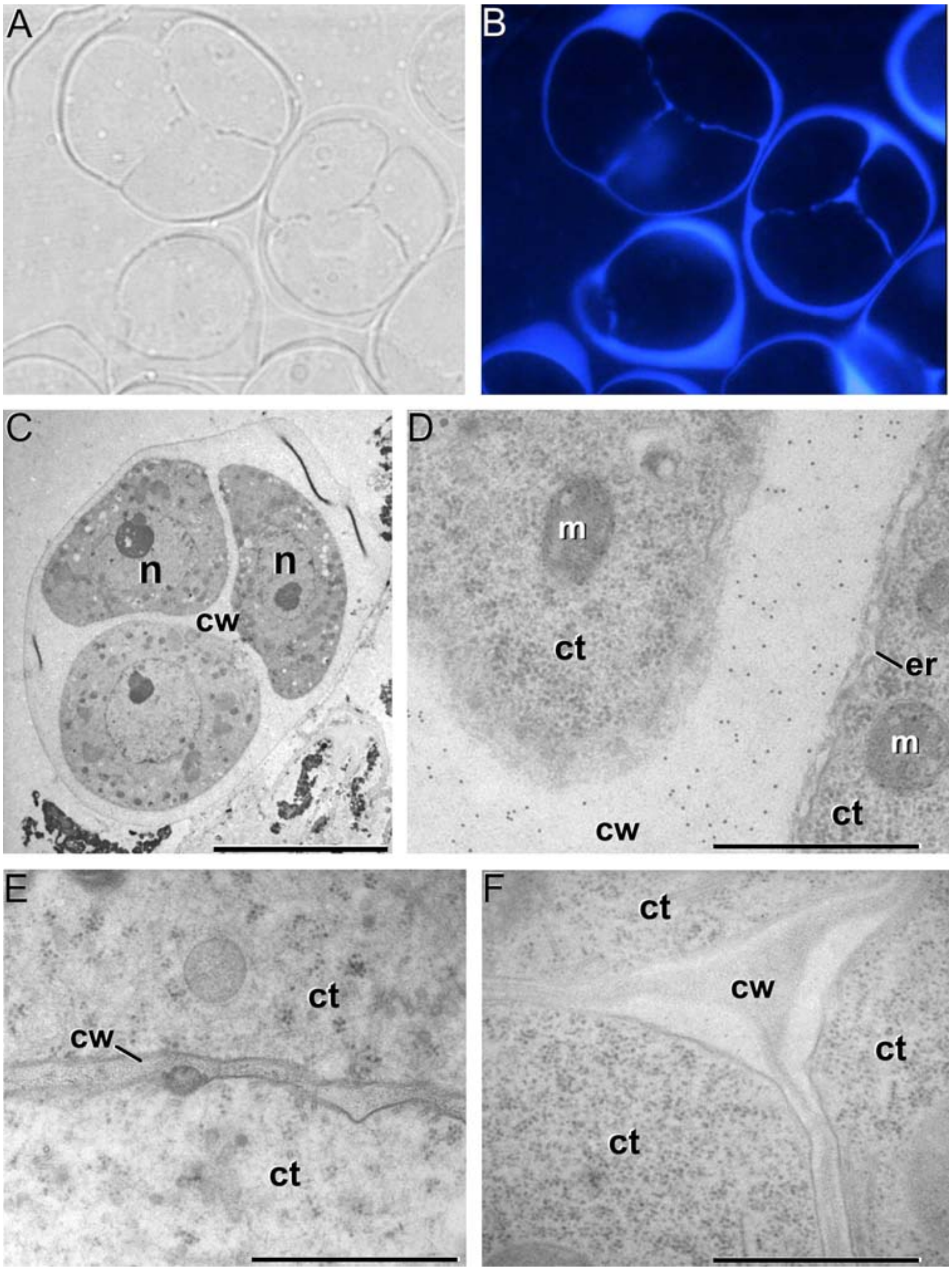

Supplementary Figure S1: Callose identification in tomato meiocytes and controls in *B. napus* cultures. A, B: DIC (A) and fluorescence (B) images of meiocytes stained with aniline blue. C, D: Immunogold labeling of meiocytes with anti-callose antibody. Note the abundant presence of gold particles in the callose-rich cell walls (cw). E, F: Controls of the anti-callose immunolocalization experiments excluding the primary antibody in two-celled

embryogenic microspores (E) and heart-shaped embryos (F). Note the absence of gold particles. ct: cytoplasm; er: endoplasmic reticulum; m: mitochondria; n: nucleus. Bars: C: 10 µm, D-F: 1 µm.

For processing of tomato meiocytes for transmission electron microscopy, tomato anthers carrying meiocytes were selected according to the criteria used in Seguí-Simarro and Nuez (2005) and processed according to Seguí-Simarro and Nuez (2007). Briefly, samples were fixed in Karnovsky fixative + 2% OsO4 for 4 h at room temperature, dehydrated in acetone series and embedded in Epon resin. Ultrathin (80 nm) sections were obtained, mounted onto copper grids, counterstained with uranyl acetate and lead citrate, and observed in a Philips CM10 transmission electron microscope. Immunogold labeling with anti-callose antibodies was performed as described in Materials and Methods.

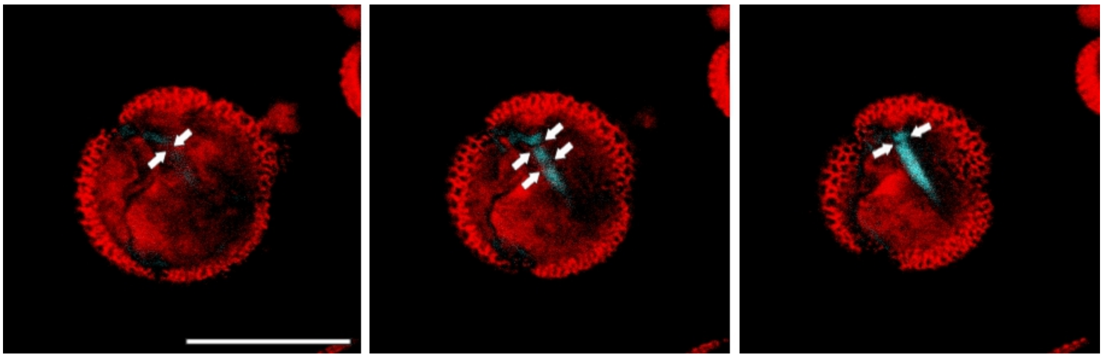

Supplementary Figure S2: Staining of *B. napus* embryogenic microspores with aniline blue and PI. Confocal slices taken at three different depths along the Z-axis. Arrows point to regions of the cell wall devoid of anilin blue staining, where PI staining (red) delineates the existence of cytoplasmic bridges connecting the daughter cells. The dark red signal at the exine corresponds to autofluorescence. Bar: 25 µm.

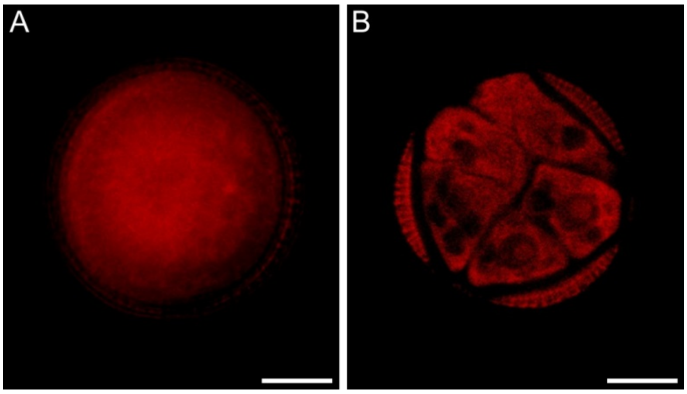

Supplementary Figure S3: Staining of *B. napus* cultured microspores with aniline blue and PI. Pollen-like structure (A) and multicellular embryogenic microspore (B) with no aniline blue staining. The dark red signal at the exine corresponds to autofluorescence. Bars: 10 µm.

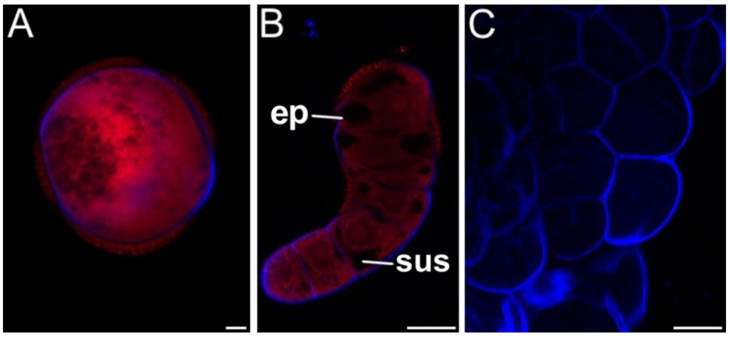

Supplementary Figure S4: Staining of *B. napus* cultured microspores with calcofluor white (blue) and PI (red). A: Pollen-like structure with cellulose staining (blue) at the intine. B: Octant suspensor-bearing MDE with cellulose staining at the outer and inner cell walls of the suspensor (sus). Note that the cell walls of the embryo proper (ep) show a barely detectable blue staining only at the outer walls. C: Transitional MDE (globular to heart-shaped) showing abundant cellulose staining in all the cell walls. The dark red signal at the exine in A and B corresponds to autofluorescence. Bars: 10 µm.

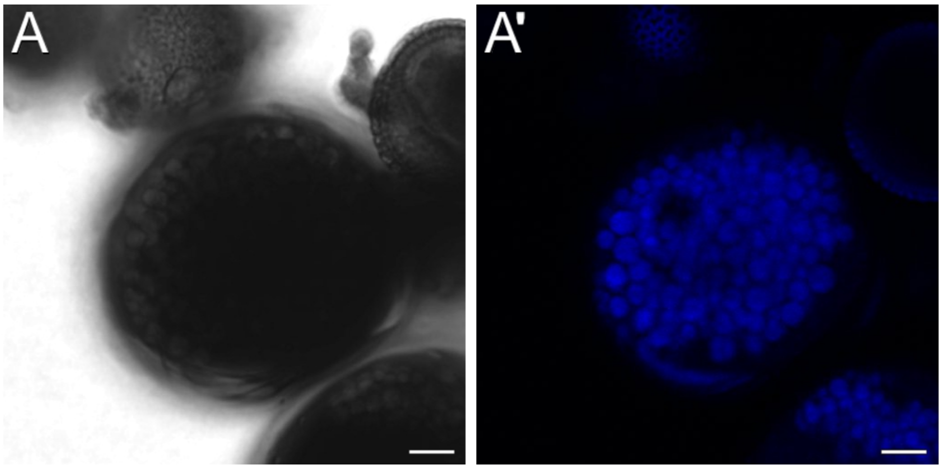

Supplementary Figure S5: Starch-rich *B. napus* pollen grains stained with aniline blue. The blue cytoplasmic spots correspond to starch granules. Although unexpected, this is not a surprising finding. Aniline blue is not a starch-specific stain, but it has a demonstrated affinity for multiple kinds of biological molecules, including not only callose but also laminarin and substituted β-1,3-glucans (Albersheim *et al.*, 2011). This is probably due to the fact that aniline blue is not a pure chemical, and together with its two main components, it may contain traces of other molecules capable of staining different carbohydrates with branching residues or links similar to those of callose. In parallel, starch granules are widely known to bind different kinds of molecules, from actin to antibodies, in a non-specific manner. Bars: 5 µm.

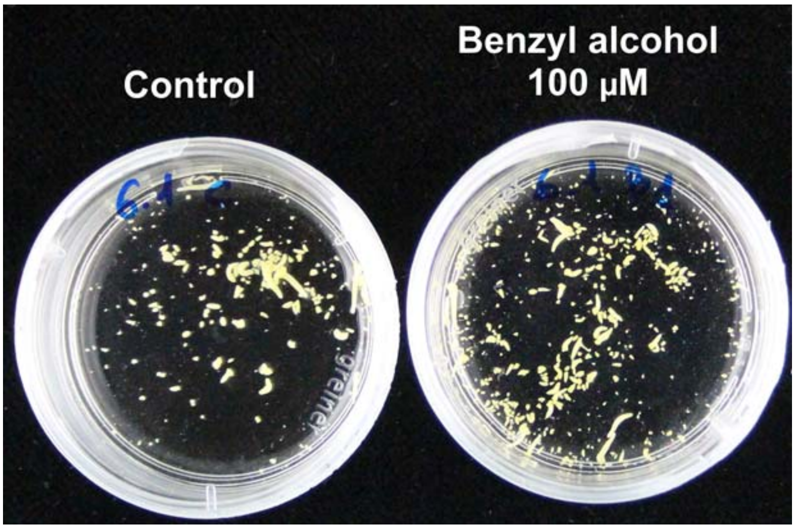

Supplementary Figure S6: BA-treated *B. napus* microspore cultures. Note the difference in the amount of embryos produced between the control dish (left) and the BA-treated dish (right).

Supplementary references

Albersheim P, Darvill A, Roberts K, Sederoff R, Staehelin A. 2011. Plant cell walls. New York, USA: Garland Science.

Seguí-Simarro JM, Nuez F. 2005. Meiotic metaphase I to telophase II is the most responsive stage of microspore development for induction of androgenesis in tomato (*Solanum lycopersicum*). *Acta Physiologiae Plantarum* **27,** 675-685.

Seguí-Simarro JM, Nuez F. 2007. Embryogenesis induction, callogenesis, and plant regeneration by *in vitro* culture of tomato isolated microspores and whole anthers. *Journal of Experimental Botany* **58,** 1119-1132.
